# Feeling What an Insect Feels

**DOI:** 10.1371/journal.pone.0108895

**Published:** 2014-10-01

**Authors:** Abdenbi Mohand Ousaid, Guillaume Millet, Sinan Haliyo, Stéphane Régnier, Vincent Hayward

**Affiliations:** 1 ISIR - Institut des Systèmes Intelligents et de Robotique, Sorbonne Universités, UPMC Univ Paris 06, UMR 7222, Paris, France; 2 CNRS, UMR 7222, Paris, France; Duke University, United States of America

## Abstract

We describe a manually operated, bilateral mechanical scaling instrument that simultaneously magnifies microscopic forces and reduces displacements with quasi-perfect transparency. In contrast with existing micro-teleoperation designs, the system is unconditionally stable for any scaling gains and interaction curves. In the present realization, the work done by the hand is more than a million times that done by a microscopic probe so that one can feel complete interaction cycles with water and compare them to what is felt when an insect leg interacts with a wet surface.

## Introduction

The cantilevered atomic force microscope [1] is an advance that can be compared with that of the optical microscope [2], since it gave access to the mechanical microworld like the microscope gave access to the optical microworld. However, unlike the optical microscope, the atomic force microscope is a scanning device and thus does not easily lend itself to be an interactive instrument: observations are made accessible to the users in the form of grids of data points that can be visualized offline.

The transformation of the atomic force microscope into an interactive instrument was previously attempted using conventional teleoperation approaches [3]–[5], where scaled position and force signals are cross-linked between ‘slave’ and ‘master’ manipulator devices [6], [7]. When treating the atomic force microscope like a slave manipulator, however, the phases of interaction where the probe tip is attracted to a sample are inherently unstable and thus inaccessible to direct human experience. In the present realization, this shortcoming was eliminated by employing an active probe that could track the entirety of tip-sample microscopic interaction curves.

Teleoperation theory shows that in conventional systems the choice of scaling gains is fundamentally limited [8], [9]. In the present design, the active probe was coupled to a novel force-feedback user interface that closely approximated a pure force generator, realizing a mechanical scaling instrument that was unconditionally stable for any scaling gains, while maintaining quasi-ideal transparency in the frequency range relevant to human interaction. During operation, the user felt as if she or he was directly interacting with an enlarged replica of the sample where the macroscopic exchange of mechanical work done by the hand was six-seven orders of magnitude larger than that taking place in the microscopic world.

**Figure 1:**
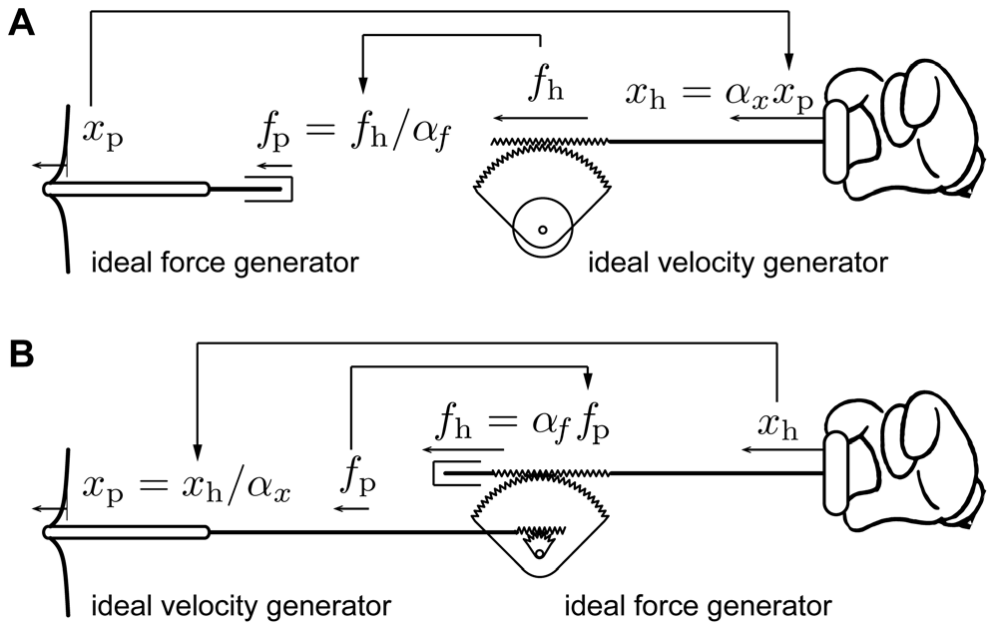
Ideal mechanical scaling instrument. (A, B) Two possible signal causality schemes. (A) The probe is an ideal force generator, it has no mass and is infinitely compliant. The handle is infinitely rigid and imposes scaled up displacements. Scaled down hand interaction forces are reflected to the probe. (B) The probe is infinitely rigid and imposes scaled down displacements to the sample. Scaled up probe interaction forces are applied to the hand through an ideal force generator.

The broad principle of an ideal mechanical scaling instrument may be described by one of the two hypothetical devices shown in Figures 1A, B. A probe interacting with a sample moves by an amount, 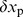, where to each displacement, 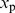, corresponds a force, 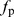, possibly not uniquely. Scaling up a microscopic interaction requires an external source of power since the work, 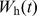, performed by the hand should be orders of magnitude larger than the work, 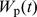, performed by the probe. If 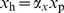 is the displacement of the handle and if 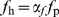 is the force applied by the hand, then 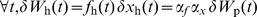, where the value of the product, 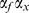, should be at least a million in micro-scale applications (1.0 µN 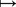 1.0 N, 1.0 µm 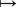 1.0 mm).

The scaled mechanical work transferred from the sample to the probe can be either generative or dissipative. In the microscopic world, such inversions are commonplace, for instance at the onset of adhesion where the probe is suddenly attracted to the sample. Considering that the work to be scaled up is at all times the product of force and displacement, two options are available to us to constrain the hand to perform a scaled-up replica of the work performed by the probe. In one option, see Figure 1A, the probe is arranged to have sufficiently high mobility — mobility is the ratio of velocity to force — in order to apply small controlled forces regardless of its movements. These probe forces are scaled-down versions of the measured forces applied by the hand onto the handle. Concomitantly, measured probe displacements are relayed to the hand through an ideal velocity generator that imposes scaled up displacements to the hand overcoming any force it applies. In the other option, see Figure 1B, the probe must have sufficiently low mobility and the measured forces of the microscopic interaction are transmitted in amplified form to the hand through a high mobility force generator. At the same time, the measured hand displacements are scaled down and imposed to the probe by an ideal velocity generator.

The two options are in principle equivalent, but in practice they are quite different. In the option of Figure 1A, a high mobility probe can be realized by a compliant cantilever with a probing tip, similar to atomic force microscope implementations. The specification of interaction forces independently from the tip movements then requires to displace the cantilever base at high speeds and with high amplitudes, which is difficult. The realization of the user interface is also difficult. The force of interaction between the hand and the handle can be confounded with spurious dynamic forces owing to the fast movements of the handle. With the option of Figure 1B, the realization of a low mobility probe is easily accomplished using a micro-positioner. However, the requirement for low mobility of the probe conflicts with that for force sensitivity. We resolved this conundrum by using feedback in an active compensation scheme to convert a high mobility, actuated probe into a low mobility probe, while preserving sensitivity. On the user side, the realization of a force generator of low mobility intended to interact with the hand is difficult because the dynamics of actuators are typically of second-order. Closed-loop control therefore requires acceleration feedback, which is hard to stabilize robustly. We solved this problem by arranging the primary force generator to have inherent first-order dynamics. The scaling down of displacements is straightforward.

**Figure 2:**
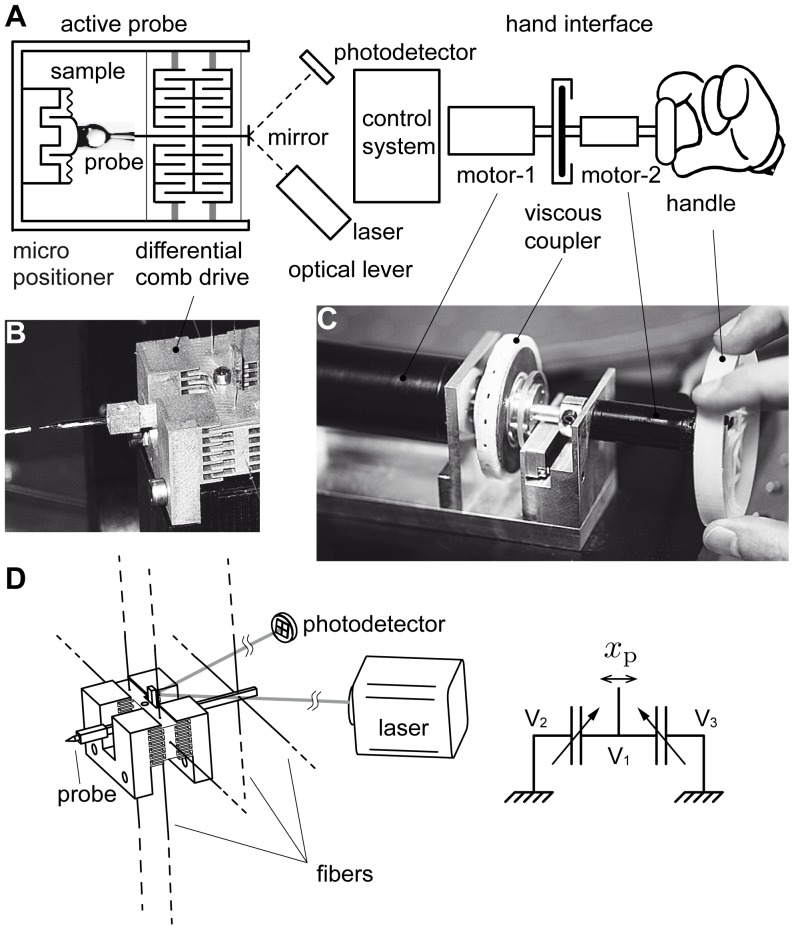
Practical realization of the second scheme. (A) Differential electrostatic comb drive with a moving armature suspended by a network of glass fibers. The probe, connected to the moving armature, interacts with a sample that is moved by a micro-positioner in response to the handle movements. The position of the probe is detected by an optical lever. The position signal is sent to the control system which returns an actuator control voltage that nulls the probe displacement. The control system drives the hand interface by servoing the velocity of motor-1, transmitting torque via a Foucault-current coupler to the handle attached to motor-2. Because the torques add on a common shaft, motor-2 can fill in the missing transients. The forces transmitted to the hand do not include the inertial forces arising from the movements of the large motor-1. (B) Physical realization of active probe. (C) Physical realization of the hand interface. (D) Detail of the fiber suspension guiding the actuator armature along rectilinear movements with high compliance. The two mechanically grounded armatures are assigned voltages 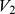 and 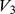 respectively and the moving armature voltage 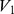. It can be shown that if 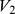 and 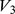 are such that 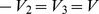, and if 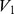 is fixed to a constant value, the actuator force, 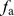, is given by 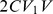, where 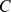 is the actuator capacitance. The actuator force is thus proportional to 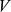 [10].

**Figure 3:**
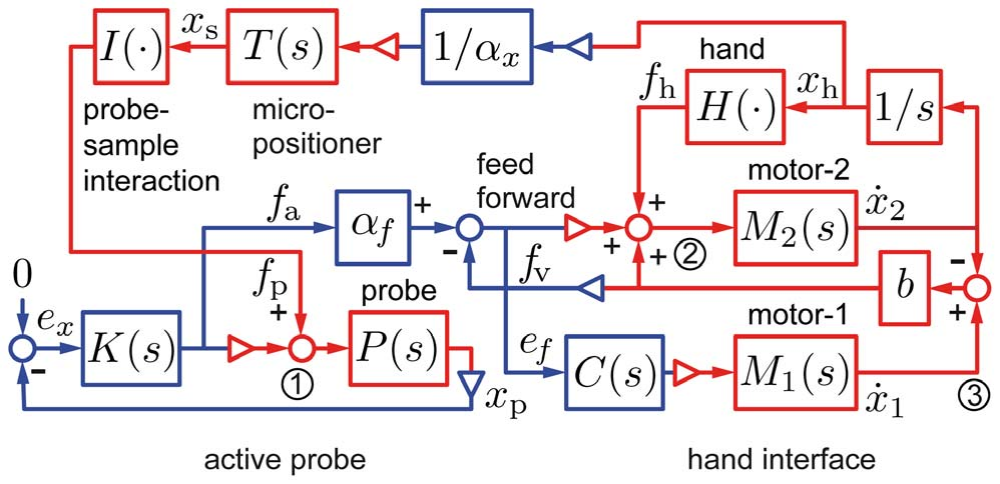
Control scheme. Computational signals and blocks are in blue and physical signals are in red. The control, 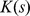, looped around the probe, 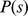, is designed to achieve high stiffness. Electrostatic forces are at all times equal to the interaction forces. The force generator operates with a velocity control, 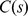, and feed-forward compensation taken from the force error signal, 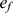. Crucially, the physical summation nodes (red) combine force signals without approximations. Nodes 1 and 2 represent Newton's second law. Node 3 represents the viscous force corresponding to the slip velocity of the coupler. Computational summation nodes are all associated to error terms.

We realized a practical mechanical scaling instrument following the option represented in Figure 2A. For brevity, in the foregoing no distinction is made between forces and torques. The feedback system of the probe comprised a differential electrostatic actuator having a linear transfer characteristic [10] and an optical lever to detect its position. The position of the sample relative to the probe was determined by a micro-positioner made of a voice-coil motor acting against an elastic suspension. The central component of the user interface, the force generator, was a viscous coupler based on the principle of Foucault currents. These non-contact devices have a near-perfect viscous behavior as long as slip velocity is below a critical value [11]. The armature was a thin disk of aluminum of low inertia and the relationship between input slip velocity and output torque was of first order. The output torque was proportional to the relative velocity between the rotor (motor-1) and the armature (motor-2 and handle). The control system diagrammed in Figure 3 was designed to provide properties closely approaching that of the ideal mechanical scaling device of Figure 1B.

## Materials and Methods

### Active probe

The probe was designed around a bipolar differential electrostatic actuator about 1.0 cm in scale generating a force proportionally to the voltage applied, see Figure 2B, D. The moving armature carrying the probe was suspended by a system of five glass fibers that provided exact kinematic guidance along one direction with no mechanical hysteresis. The optical lever comprised an external laser source, a mirror attached to the carrier and an external, four quadrant photodiode. The system exhibited a natural angular frequency of 64 rad/s and a stiffness of ∼4.0 N/m. In the foregoing, 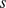 is the Laplace variable.

Feedback control, shown in Figure 3 (left feedback loop), overcame the limitations related to passive sensors by stiffening the probe actively. The probe-sample interaction, 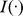, gave rise to a force, 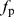, that tended to deflect the probe to a measured position, 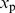. The compensator, 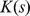, forced the probe, 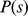, to null the position error, 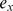. The known actuator force, 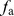, then was, within the controller bandwidth, an accurate replica of the interaction force, 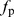, acting as an input disturbance, 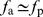. The range of forces that can be handled by the active probe was ±400 µN. With a resolution of 0.4 µN, measurements could be made with a dynamic range of three orders of magnitude and the sensitivity was well within the range of capillary forces.

In Figure 3, node 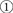 represents the summation of the forces acting on the moving armature of the actuator. Equilibrium was achieved in a wide range of conditions owing to the robust stability properties of the feedback within the control bandwidth, and when it was smaller than the actuator saturation level. The position feedback controller was optimally designed using 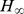 procedures [12]. The numerical expressions of the probe and of the controller transfer functions are given in the supporting information, file Text S1, equations (1) and (2). For accuracy, all design procedures for the control system (Figure 3, continuous time) were carried out in the discrete time domain with a sampling period of 1.0 ms.

The sample was displaced by a micro-positioner to a position 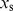. Its transfer function, 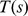, could be for all practical purposes taken to be unity, 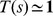.

### User interface

The force-feedback user interface comprised two stages as shown by Figures 2A, C. It had a large motor (motor-1) that produced torque transfered to a small motor (motor-2) through a Foucault-current viscous coupler. The small motor was rigidly connected to a handle having very low inertia. The large motor (Maxon RE-35-273754) drove the inductor of the coupler. The inductor was fitted with sixty 20 mm^2^ neodymium magnets. The rotor was a 1 mm thick, 52 mm outer diameter aluminum annulus. The shaft positions of the two motors were detected by high-resolution digital encoders (MicroE Mercury M1800). The coupler's viscous coefficient was 8.7 10^−4^ N m s 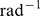.

A velocity feedback controller, 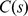 (see Figure 3 right feedback loop), set the velocity of motor-1, 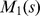, to achieve a desired force output, 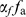. The controller was implemented as a discrete-time, polynomial pole-placement compensator that ensured robust regulation and tracking performance [13]. The numerical expressions of the controller and the reference model are given in supporting information, file Text S1, equations (3) to (6).

Since the bandwidth of human voluntary movements was well within the bandwidth of the velocity control loop, the dynamics of motor-1 was entirely eliminated from the user's haptic experience [14]. Nevertheless, the force signal to be reproduced could contain fast components that the velocity servo loop could not track. To fill-in the transients, a feed-forward path was provided through the low-power, high-precision motor-2 (Maxon RE-16-118698). Because this motor had negligible friction and inertia, unwanted forces could be kept below human detection thresholds, achieving quasi-perfect transparency.

The quantitative evaluation of the transparency of the interface was reported in [14]. It was designed to operate at the limits of human sensory detection performance with an equivalent inertia of 5.2 g and a friction of 3.7 mN at the finger contact with the manipulandum. The improved transparency of the interface allowed users to detect details that were ten times smaller in magnitude than those detected when using a conventional design.

The summation node 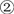 in Figure 3 represents the sum of all forces that acted on motor-1, namely, the low-frequency viscous force arising from the coupler slip velocity (node 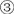), 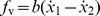, the force applied by the user's hand, 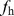, and the transient feed-forward error signal, 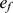. The position of the handle, that is of motor-2, 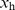, was scaled by a factor, 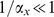, to determine the position, 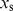, of the sample.

### System closed-loop stability

It can be shown that such a system was stable for any bounded value of the scaling factors, 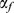 and 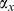. Closed-loop stability was unconditional for any nonlinear interactions between the probe and sample, 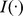, and between the handle and hand, 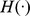. In the worst case when no dissipation was present, a sufficient proof of stability was achieved through the application of the Llewellyn's absolute stability criterion [15], [16]. Alternatively, it can be verified that all the system's poles had a negative real part and the real part of the transfer function was positive over the entire bandwidth, from DC to 64 rad/s. Passivity of the entire interaction chain was then guaranteed given that the interconnection of linear time invariant passive systems yields a passive system. Since 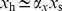, and since 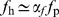 up to the open-loop dynamics of the small motor, we have 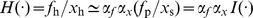 which means that the user felt a nearly exact replica of the probe-sample interaction dynamics scaled by a factor, 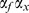, without any gain-stability trade-off.

## Results

**Figure 4:**
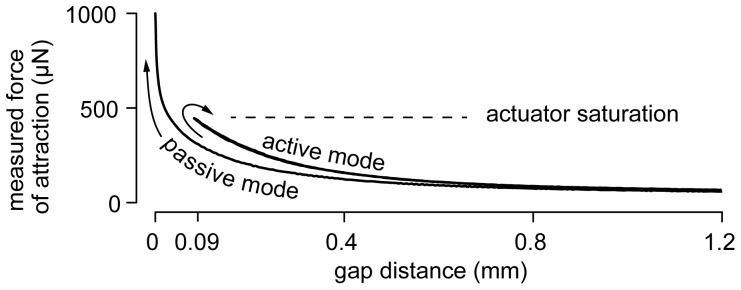
Active vs passive probe proving experiment. A sewing needle tip interacts with a magnet. In passive mode, owing to the high compliance of the probe, the interaction becomes unstable when the gap becomes smaller than 0.8 mm, whereas, in active mode, the interaction remains tractable down to 90 µm, until the actuator saturates. In the active mode, approach and retraction curves fall on top of each other, denoting absence of hysteresis, and the gap may be arbitrarily small, subject to actuator saturation (±400 µN). In the passive mode, the measurement is erroneous owing to uncontrolled probe dynamics.

The bilateral capability of the system was first tested by exploring the interaction curve between a small fragment of a magnet and the point of a sewing needle. When the feedback loop was inactive, the system behaved like an atomic force microscope cantilever probe, exhibiting an unstable jump-to-contact motion once the attraction force exceeded the elastic force restraining the probe, see Figure 4. In contrast, the active probe could track the entirety of the magnetic interaction force regardless of the proximity to the sample, eliminating measurement hysteresis and the blind region, up to actuator saturation.

**Figure 5:**
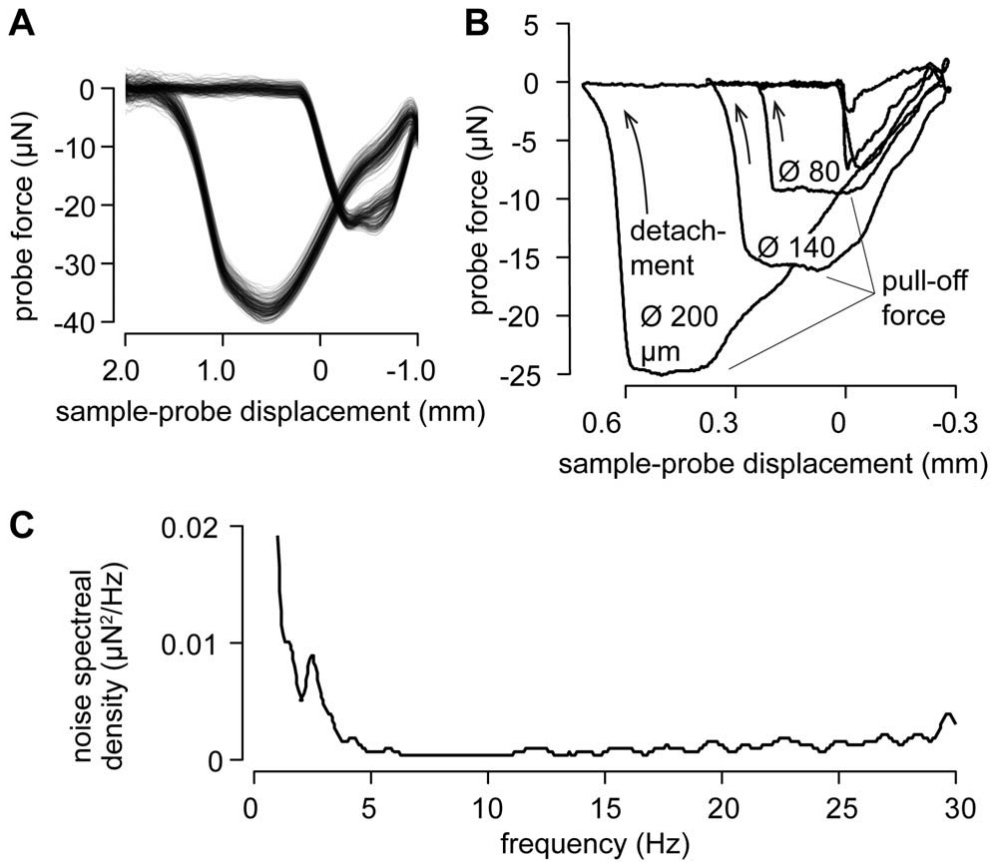
System performance. (A) One hundred and seventy-five repetitions of a cyclical interaction with the same water droplet at a rate of one per second showing excellent repeatability. The measurements differ by a small drift term owing to thermal fluctuations and/or water evaporation. (B) The pull-off force is by and large proportional to the probe diameter. (C) Noise spectral density of the unloaded force sensor.

The repeatability, the sensitivity, and the noise performance of the system were then assessed. One hundred and seventy-five cyclical interactions with the same droplet of water at a rate of one per second showed that the measurements were repeatable, see Figure 5A, differing only by a small drift from cycle to cycle. If the system was sensitive to capillary forces, following the Young-Laplace law, the force measurements at pull-off should depend on the probe diameter according to 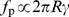 where 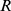 is the probe radius and 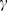 the surface tension. The interactions reported in Figure 5B showed that it was indeed the case for probe diameters of 80, 140, and 200 µm. Finally the noise performance is reported in Figure 5C.

**Figure 6:**
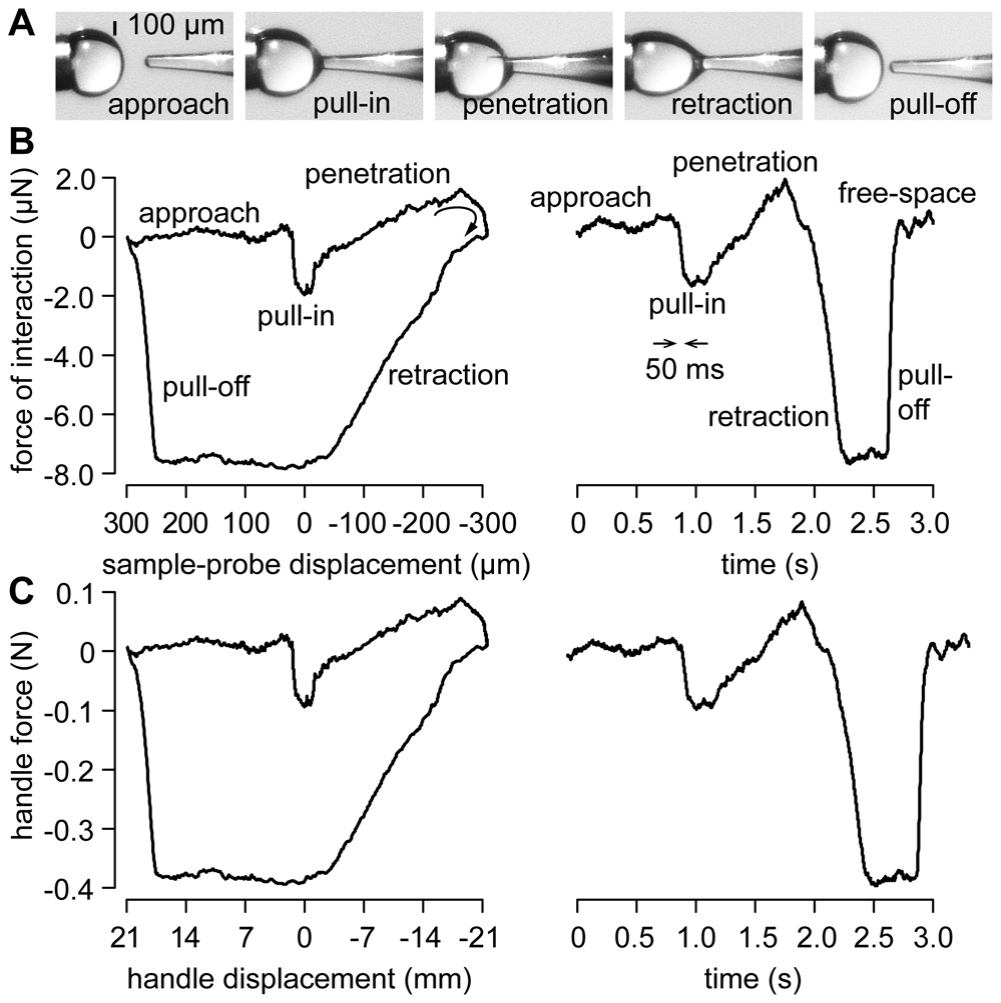
Interaction of a glass probe with a droplet of water. (A) Main interaction phases. (B) Evolution of the probe force, 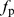, as a function of probe displacement and as a function of time. In the approach phase, the force signals are reduced to noise. At the instant of contact, the probe is suddenly attracted by the droplet owing to capillary forces. Penetrating the droplet corresponds to a gradual increase of the interaction force. Retraction inverts the sign of the force and the interaction exhibits large hysteresis until the contact snaps off when exceeding a given deflection. (C) Force felt by the hand, that is 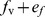, that accurately replicates the microscopic interaction.

The system was then validated by probing droplets of water with a glass micropipette having a tip diameter of ∼80 µm as illustrated by Figure 6A. The different phases of the interaction were felt by the experimenter as if she or he was directly touching the droplet, but with the difference that the mechanical work done by the hand of the experimenter was at all times 3.5 million times larger than that of the probe as can be seen from the signals shown in Figure 6B, C. When first touching the droplet surface, the experimenter felt a sudden jolt, a step of 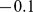 N in 50 ms visible in Figure 6C, at the end of the approach phase, corresponding to the formation of the meniscus at the instant of ‘pull-in’. Subsequently, the interaction force increased with the penetration distance. This increase was likely to be due to a combination of the enlargement of the meniscus and of the ‘piston’ section responding to the positive hydrostatic pressure inside the droplet. A reversed movement during the retraction phase corresponded to a strong adhesive force that increased to 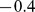 N, that is −8.0 µN in reality, see Figure 6B, C, where the force becomes negative, until the contact snapped when the droplet was deflected by −250 µm from the initial contact at distance zero.

**Figure 7:**
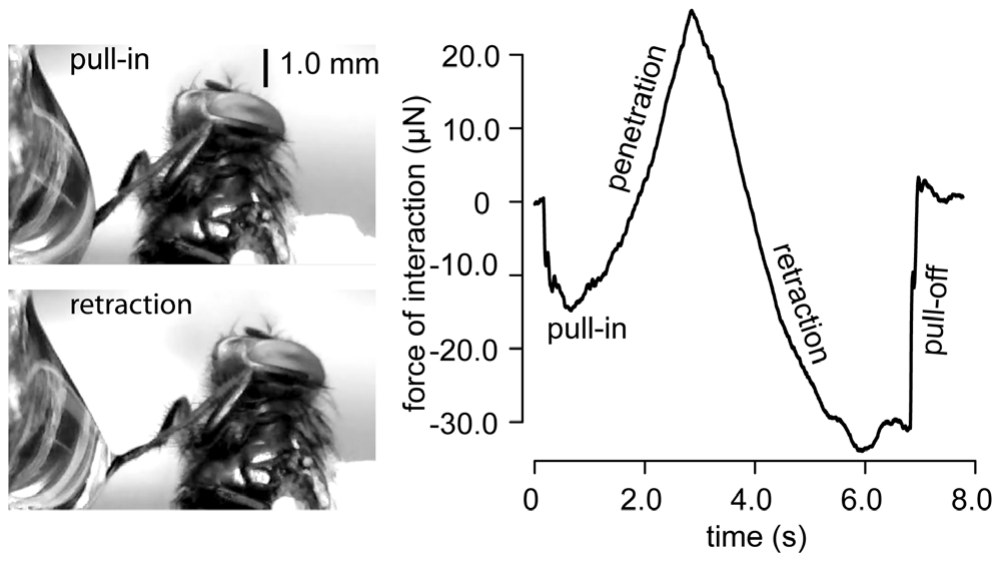
Interaction of an insect leg with a droplet of water. Similar maneuver as in Figure 6 but using a house fly leg as a probe. While the fly's leg is of similar size than the glass probe, interaction forces are ten fold larger with the insect leg than with the glass probe.

The nature and magnitude of forces during this interaction are representative of an insect's leg adhering to a substrate [17], [18]. A similar maneuver executed with the leg of a house fly, as reported by Figure 7, let the experimenter directly experience the fact that the multi-scale structure of the leg of an insect greatly magnified the interaction forces, although the leg was actually smaller than the glass probe. The file Video S1 shows a live ant interacting with a water droplet where the magnified interaction forces experienced by the ant are felt by a human observer.

## Discussion

The mechanical scaling instrument gave direct access to micro-scale phenomena previously not felt by humans, although they could be seen through a microscope and measured through conventional instruments. The probe had very low mobility compared to the sample but high force sensitivity and the handle had very high mobility compared to the hand and could eliminate most spurious forces from the interaction. These combined properties provided nearly perfect transparency without any stability trade-off so that scaling gains could be made arbitrarily high, subject only to saturation. Other practical limits were due to the inertia of the probe, which could be reduced through miniaturization, and to sensing noise in the optical lever which could be replaced by an interferometric measurement technique.

The availability of interactive manipulation at the micro-scale enables many immediate applications ranging from the handling and probing of biomaterials to the assembly of microstructures. Because operation is achieved through feedback control, this technique lends itself naturally to coupling manual control with automation, similarly to many successful applications of robotic technologies, such as surgical robotics or robotic space exploration. Paths for improvements include miniaturization of the probe and expansion of the system to multiple degrees of freedom.

## Supporting Information

Data S1 **Raw data underlying the findings.** This compressed archive contains all the experimental data related to our study. It is composed of a Guidelines file (pdf) and fourteen data files in Matlab format (mat). Those data are used within the manuscript in Figs. 4, 5A, 5B, 5C, 6B, 6C, and 7.
(ZIP)

Text S1 **Numerical expressions of the system plant and controllers.**
(PDF)

Video S1 **Interaction between an ant and a droplet.** The video shows a live ant interacting with a water droplet. The magnified interaction forces experienced by the ant are felt by a human operator.
(AVI)
