# Supplementary material for: Feeling What an Insect Feels
Source: PLoS One. 2014 Oct 1;9(10):e108895. doi: 10.1371/journal.pone.0108895 (PMC4182749; doi:10.1371/journal.pone.0108895)
Supplement: Data S1 — Raw data underlying the findings. This compressed archive contains all the experimental data related to our study. It is composed of a Guidelines file (pdf) and fourteen data files in Matlab format (mat). Those data are used within the manuscript in Figs. 4, 5A, 5B, 5C, 6B, 6C, and 7. (ZIP) [file pone.0108895.s001.zip › Experimental data/Data_Guidelines.pdf]

Data guidelines

This document gives some guidelines about the content of the data available as supplemental files. All data have Matlab format data (*.mat).

1. Active vs passive probe proving experiment (Figure 4)

"magnetic_force_closed_loop_Fig4" and **"magnetic_force_open_loop_Fig4"** are related to the magnetic force measurement. They were used to plot Figure 4. The first column of each file corresponds to position while the second column corresponds to measured force.

2. System performance (Figure 5)

Figure 5A

"data_sensor_reliability_pos_Fig5A" is related to one hundred and seventy-five repetitions of a cyclical interaction with the same water droplet at a rate of one per second. The data were used to plot the curve given in Figure 5A. "**data_sensor_reliability_time**" is related to the previous data (**data_sensor_reliability_pos_Fig5A**) experience where the first column corresponds to time.

Figure 5B

"**Int_force_probe80**", "**Int_force_probe140**" and "**Int_force_probe200**" are related to three approach-retract cycles of a probe over a water droplet using three probes with different diameters, respectively 80 µm, 140 µm and 200 µm. The first column of each data represents the relative position between probe and droplet, while the second column represents the interaction force between the probe and the droplet. They were used to plot Figure 5B.

Figure 5C

"Unloaded_force_sensor" is related to the force measured when the sensor is unloaded. The first column is time where the second column represents force, in other words, it represents noise. "**Noise_spectral_density**" represents the noise spectral density of the unloaded force sensor (data given by **Unloaded_force_sensor**). The first column is frequency and the second column represents the noise spectral density.

3. Interaction of a glass probe with a droplet of water (Figure 6)

Figure 6B

"**Int_force_droplet_probe_pos**" is related to the interaction force measurement of a droplet with a probe of diameter of 80 µm. The first column is the relative position between the droplet and the probe tip, while the second is the interaction force measured between the probe and the droplet. These data were used to plot Figure 6B.1.

"**Int_force_droplet_probe_time**" is related to same experience where the first column represents time. These data were used to plot Figure 6B.2.

Figure 6C

"**Handle_force_pos**" is related to the same experience where the second column represents the force felt by the user (the force at the circumference of the haptic interface handle). These data were used to plot Figure 6C.1. "**Handle_force_time**" represents the same experience where the first column is time. They were used to plot figure 6C.2.

4. Interaction of an insect leg with a droplet of water (Figure 7)

"insect_force_interaction_Fig7.mat" is related to the measurement of the interaction force of a house fly leg with a water droplet. These data were used to plot the curve given in Figure 7. The first column corresponds to time while the second column represents the interaction force.
