# Supplementary material for: Feeling What an Insect Feels
Source: PLoS One. 2014 Oct 1;9(10):e108895. doi: 10.1371/journal.pone.0108895 (PMC4182749; doi:10.1371/journal.pone.0108895)
Supplement: Text S1 — Numerical expressions of the system plant and controllers. (PDF) [file pone.0108895.s002.pdf]

Numerical expressions of the system plant and controllers

1. Numerical expressions of the probe and the controller transfer functions, $P(z)$ and $K(z)$:

$$P(z) = \frac{10^{-5}(6.5\,z + 6.549)}{z^2 - 1.995\,z + 0.9994} \quad (1)$$

$$K(z) = \frac{2.232 + 11.65\,z - 29.95\,z^2 + 16.11\,z^3}{0.0005445 - 0.7045\,z + 2.371\,z^2 - 2.667\,z^3 + z^4} \quad (2)$$

2. Numerical expression of the force feedback interface controller:

   (a) Polynomials $R(z^{-1})$, $S(z^{-1})$ and $T(z^{-1})$:

$$R(z^{-1}) = 2.1170 \quad (3)$$

$$S(z^{-1}) = 1 - 0.501z^{-1} \quad (4)$$

$$T(z^{-1}) = 0.03289 - 0.04792\,z^{-1} + 0.01783\,z^{-2} \quad (5)$$

   (b) Reference model:

$$H_{\mathrm{m}}(z^{-1}) = \frac{0.09279\,z^{-1} + 0.06896\,z^{-2}}{1 - 1.245\,z^{-1} + 0.4066\,z^{-2}} \quad (6)$$
